# Macular Telangiectasia Type 2: A Classification System Using MultiModal Imaging MacTel Project Report Number 10

**DOI:** 10.1016/j.xops.2022.100261

**Published:** 2022-12-08

**Authors:** Emily Y. Chew, Tunde Peto, Traci E. Clemons, Ferenc B. Sallo, Daniel Pauleikhoff, Irene Leung, Glenn J. Jaffe, Tjebo F.C. Heeren, Catherine A. Egan, Peter Charbel Issa, Konstantinos Balaskas, Frank G. Holz, Alain Gaudric, Alan C. Bird, Martin Friedlander

**Affiliations:** 1 Division of Epidemiology and Clinical Applications, National Eye Institute, National Institutes of Health, Bethesda, Maryland; 2 Centre for Public Health, Queen’s University, Belfast, United Kingdom; 3 The Emmes Corporation, Rockville, Maryland; 4 Jules Gonin Eye Hospital, University of Lausanne, Lausanne, Switzerland; 5 Department of Ophthalmology, St. Franziskus Hospital Muenster, Muenster, Germany; 6 NIHR Biomedical Research Centre at Moorfields Eye Hospital NHS Foundation Trust and UCL, Institute of Ophthalmology, London, United Kingdom; 7 Department of Ophthalmology, Duke University, Durham, North Carolina; 8 Oxford Eye Hospital, Oxford University Hospitals NHS Foundation Trust, Oxford, United Kingdom; 9 Nuffield Laboratory of Ophthalmology, Nuffield Department of Clinical Neurosciences, University of Oxford, Oxford, United Kingdom; 10 Department of Ophthalmology, University of Bonn, Bonn, Germany; 11 Department of Ophthalmology, Université Paris Cité. APHP, Hôpital Lariboisière, Paris, France; 12 The Lowy Medical Research Institute and the Department of Molecular Medicine, The Scripps Research Institute, La Jolla, California

**Keywords:** Macular telangiectasia type 2, Classification, Neurovascular degeneration, Classification and Regression Trees (CART), Machine learning, BCVA, best-corrected visual acuity, BLR, blue light reflectance, CART, Classification and Regression Trees, CF, color fundus, EZ, ellipsoid zone, FAF, fundus autoflorescence, FLIO, fluorescence lifetime imaging ophthalmoscopy, MacTel, macular telangiectasia type 2, NHOR, natural history observation registry, NHOS, natural history observation study, OCTA, OCT angiography, SD-OCT, spectral domain-OCT, VA, visual acuity

## Abstract

**Purpose:**

To develop a severity classification for macular telangiectasia type 2 (MacTel) disease using multimodal imaging.

**Design:**

An algorithm was used on data from a prospective natural history study of MacTel for classification development.

**Subjects:**

A total of 1733 participants enrolled in an international natural history study of MacTel.

**Methods:**

The Classification and Regression Trees (CART), a predictive nonparametric algorithm used in machine learning, analyzed the features of the multimodal imaging important for the development of a classification, including reading center gradings of the following digital images: stereoscopic color and red-free fundus photographs, fluorescein angiographic images, fundus autofluorescence images, and spectral-domain (SD)-OCT images. Regression models that used least square method created a decision tree using features of the ocular images into different categories of disease severity.

**Main Outcome Measures:**

The primary target of interest for the algorithm development by CART was the change in best-corrected visual acuity (BCVA) at baseline for the right and left eyes. These analyses using the algorithm were repeated for the BCVA obtained at the last study visit of the natural history study for the right and left eyes.

**Results:**

The CART analyses demonstrated 3 important features from the multimodal imaging for the classification: OCT hyper-reflectivity, pigment, and ellipsoid zone loss. By combining these 3 features (as absent, present, noncentral involvement, and central involvement of the macula), a 7-step scale was created, ranging from excellent to poor visual acuity. At grade 0, 3 features are not present. At the most severe grade, pigment and exudative neovascularization are present. To further validate the classification, using the Generalized Estimating Equation regression models, analyses for the annual relative risk of progression over a period of 5 years for vision loss and for progression along the scale were performed.

**Conclusions:**

This analysis using the data from current imaging modalities in participants followed in the MacTel natural history study informed a classification for MacTel disease severity featuring variables from SD-OCT. This classification is designed to provide better communications to other clinicians, researchers, and patients.

**Financial Disclosure(s):**

Proprietary or commercial disclosure may be found after the references.

Macular telangiectasia type 2 (MacTel) is a rare retinal neurodegenerative disease affecting a specific retinal area, the “MacTel area”^1^, ^2^, ^3^ and is usually associated with typical vascular changes. Vision loss initially occurs in the temporal paracentral macula, which leads to binasal scotomas. This may later involve most of the MacTel area, including the center of fovea.,^4^ Consequently, visual dysfunction primarily affects the patients’ reading ability, but later more widespread loss of visual acuity (VA) may occur.^5^^,^^6^

Since 2005, the MacTel Research Group, sponsored by the Lowy Medical Research Institute, has studied this disease with a worldwide network of clinical sites in collaboration with several basic science laboratories. The goals of this research endeavor, known as the MacTel Project (https://www.lmri.net/mactel/the-mactel-project/), were to elucidate the pathogenesis, to develop potential outcome measures for clinical trials, and to identify and test treatments for MacTel. To achieve these goals, an international multicentered natural history study using multimodal imaging was conducted to better characterize the diseases, evaluate risk factors, and determine the rates for progression of MacTel disease.

Another goal of this research group was to develop an up-to-date classification of the disease, incorporating image analysis results from novel image modalities. These newer imaging modalities are particularly relevant as the previous classification system was developed by Dr J. Donald Gass^7^ (Table 1), prior to the use of the spectral domain (SD)-OCT and fundus autofluorescence (FAF) which both show characteristic macular changes and are now widely available in clinical practice. The use of these imaging modalities has contributed significantly to the current understanding of MacTel, which had previously been considered a vascular disease. The motivation for developing a new classification system was to capture the structural changes found in the OCT of MacTel, admittedly the most informative of the more recent imaging modalities, while not detracting from the use of both conventional imaging modalities and from the clinical utility of blue light reflectance (BLR) imaging.^8^^,^^9^

**Table 1:** Gass & Blodi Classification of Macular Telangiectasia Type 2∗

1.	No obvious abnormality. early fluorescein shows minimal leakage and mild staining temporally
2.	**Graying of perifoveal retina, minimal or no telangiectatic vessels**: early fluorescein shows outer capillary network temporally
3.	**Blunted & right-angle veins**: fluorescein (stereo) shows unusual capillary dilation and permeability in outer retina
4.	**Pigmentation**: often associated within the blunted tips of right-angle veins.
5.	**Subretinal neovascular**: biomicroscopic and fluorescein evidence of neovascularization

∗ Ophthalmology. 1993 Oct;100(10):1536-46.

A revised classification using all imaging modalities is likely to facilitate a better description of the grades and progression of MacTel as well as communications between clinical and basic science researchers. These analyses may provide more insights on clinically relevant grades for future clinical trials. A classification system could also provide progression data to better inform and guide both the treating clinicians and patients on the severity level of disease. The purpose of this report is to present the analyses using the data from clinical studies performed in the MacTel Project to construct a revised classification system of MacTel.

## Methods

### MacTel Project

Different cohorts with MacTel provided data for the development and validation of this classification. They consist of the natural history observation study (NHOS—2005–2015)^10^ and the natural history observation registry study (NHOR—2010 to present). There was overlap of participants in these 2 studies, especially for those participants who have ≥ 5 years of follow-up from the NHOS and were transferred over to the NHOR. Each participating clinical site obtained approval from their institutional review board or independent ethics committee for the protocol and each participant provided written informed consent. The research was conducted in accordance with the Declaration of Helsinki and where applicable, the study complied with the Health Portability and Accessibility Act.

### NHOS (2005–2015)

The natural history of MacTel was studied in an international multicenter prospective observational study designed to evaluate the structural and functional changes associated with MacTel over ≥ 5 years of follow-up. Seven countries with 22 clinical sites were involved. Each participant who was ≥ 18 years of age enrolled into the Natural History Study after a diagnosis of MacTel was confirmed on clinical examination at the study sites based upon on stereoscopic color fundus (CF) photographs, OCT (which initially included the time-domain OCT, but by most of the follow-up, SD-OCT was the common instrument), fluorescein angiography, and FAF images which were graded by the Fundus Reading Center at Moorfields Eye Hospital, London, United Kingdom. At baseline and annual study visits, multimodal imaging and best-corrected visual acuity (BCVA) measured by trained examiners using a standardized protocol and the logarithm of the minimum angle of resolution ETDRS VA charts (scores range from 0 to 100) were obtained.

The standardized imaging involved a 30° stereoscopic field centered on the fovea that included color, red-free and fluorescein angiographic images of the retina that were captured on digital fundus cameras. Blue light FAF images were obtained using Heidelberg Retina Angiograph II or Heidelberg Spectralis SLO(+OCT) scanning laser ophthalmoscope systems (Heidelberg Engineering Inc). Spectral domain-OCT volume scans were obtained with the Heidelberg Spectralis OCT systems (Heidelberg Engineering Inc), or with the Cirrus SD-OCT 4000 systems (Carl Zeiss Meditec).

### NHOR

From 2010 to present, the MacTel Project conducted a registry of potential participants for clinical trials. Participants with a clinical diagnosis of MacTel were recruited for 1 study visit examination which included a comprehensive eye exam and the required ophthalmic imaging as previously described for the natural history study. This cohort is followed with annual telephone interviews.

### Fundus Reading Center at Moorfields Eye Hospital

The Moorfields Eye Hospital Reading Center at Moorfields Eye Hospital National Health Service Foundation Trust, London, United Kingdom, evaluated all the study images using established standardized protocols. All anonymized images were submitted to the Moorfields Reading Centre using a safe transfer protocol. The images were uploaded to their respective image viewing software. Those subjects deemed to have MacTel following a clinician adjudication process (A.C.B., T.P., C.A.E., F.B.S., T.F.C.H.) were released to the grading queue. Image grading was completed by trained and certified personnel (T.P., F.B.S., I.L.) using a prespecified protocol and data were entered into the MacTel Study’s database. Each image was evaluated independently without the knowledge of the fellow eye or prior study visit gradings. Clinician adjudication took place when the graders required further input and the clinician decision was taken as final. This process resulted in the agreed grading results for all imaging modalities to be uploaded to the MacTel study database in order to integrate with the rest of subject related data. At no time did the graders have access to any other medical information but the images. Agreement on grading characteristics was moderate to substantial for all fields.

The reading center personnel graded for MacTel features on the CF photographs, fluorescein angiograms, OCTs, and FAF images. Color fundus photograph were assessed for transparency in the perifoveal retina, pigment epithelial changes, and subretinal neovascularization. The fluorescein angiographic images were graded for abnormal vessels temporal to the fovea and subsequently in the perifoveal capillaries. The fluorescein angiography images were also graded for dilation of outer retinal vessels and the deep hyperfluorescence usually at the level of the retinal pigment epithelium or outer retina. OCT images were graded for central retinal thinning and inner and outer retinal cavities, which do not correspond to changes on fluorescein when these fluorescein angiographic images are superimposed on an “*en face*” view of the OCT. The presence of the ellipsoid zone (EZ) break on OCT and the location of the break in relation to the center of the fovea were graded on the OCT images. The presence and the position of the EZ break were also evaluated. Linear measurement was made in the horizontal B-scan closest to the foveal center. Other measures were made later using an “*en face*” methodology to determine the area of the EZ break for phase 1 clinical trial and other studies. This area of EZ break correlated with VA changes and visual field loss, as demonstrated both in the MacTel Project and other academic centers.^11^^,^^12^ Additional OCT hyper-reflectivity was also graded. This was defined as hyper-reflective lesions that were as bright as the retinal pigment epithelium layer of the OCT, which may be linear or appear as mounds that emanate from the retinal pigment epithelium and extend past the external limiting membrane of the retina.^13^, ^14^, ^15^, ^16^, ^17^ Fundus autofluorescence images were graded for the typical pattern of hyperfluorescence or the lack of masking at the central macula, as the macular pigment has been noted to be absent in MacTel.^18^^,^^19^ In addition, the right-angled vein^20^ and the black hyperpigmentation^21^, ^22^, ^23^ along the retinal vessels seen on CF photographs are also seen particularly well on the FAF image.

### Statistical Analyses

The Classification and Regression Trees (CART)^24^ is a predictive algorithm used in machine learning. The CART algorithm is nonparametric and can be used for any type of data. The CART methodology uses recursive partitioning to split the data into several groups based on values of predictor variables that create the best homogeneous group when splitting the data. To appropriately conduct CART the dataset was split into 2 sets, a training set and validation set. The training data set used to build this decision tree model was based upon the right eyes of the MacTel participants in the natural history study while the validation data set used to determine the appropriate tree size needed to achieve the optimal final model was based on the left eyes of MacTel participants. Recursive partition was conducted using dichotomized splits (presence or absence). Regression models that used a least square method created a decision tree to classify eyes into different categories. The description of the resulting decision tree developed using CART for these analyses is found in the Supplementary Information on Methods (Supplementary Materials available at www.aaojournal.org).

We conducted further validation analyses on the final classification to assess disease progression. For those participants with available follow-up data, the relative risk of progression over time for progression to ≥ 5 letter loss, ≥ 10 letter loss, and 1 or 2 step progression from baseline were determined using the Generalized Estimating Equation. Analyses were also conducted for progression over time to the more severe end of the scale.

The variables considered for analyses included the various features graded by the centralized reading center using the multimodal imaging methods. The primary target of interest for the algorithm development by CART was the change in BCVA at baseline for the right eye and for the left eye. These analyses using the algorithm were repeated for the BCVA obtained at the last study visit of the natural history study for the right eye and for the left eye.

### Reading Center Variables Evaluated in the CART Analyses

**Figure 1:**
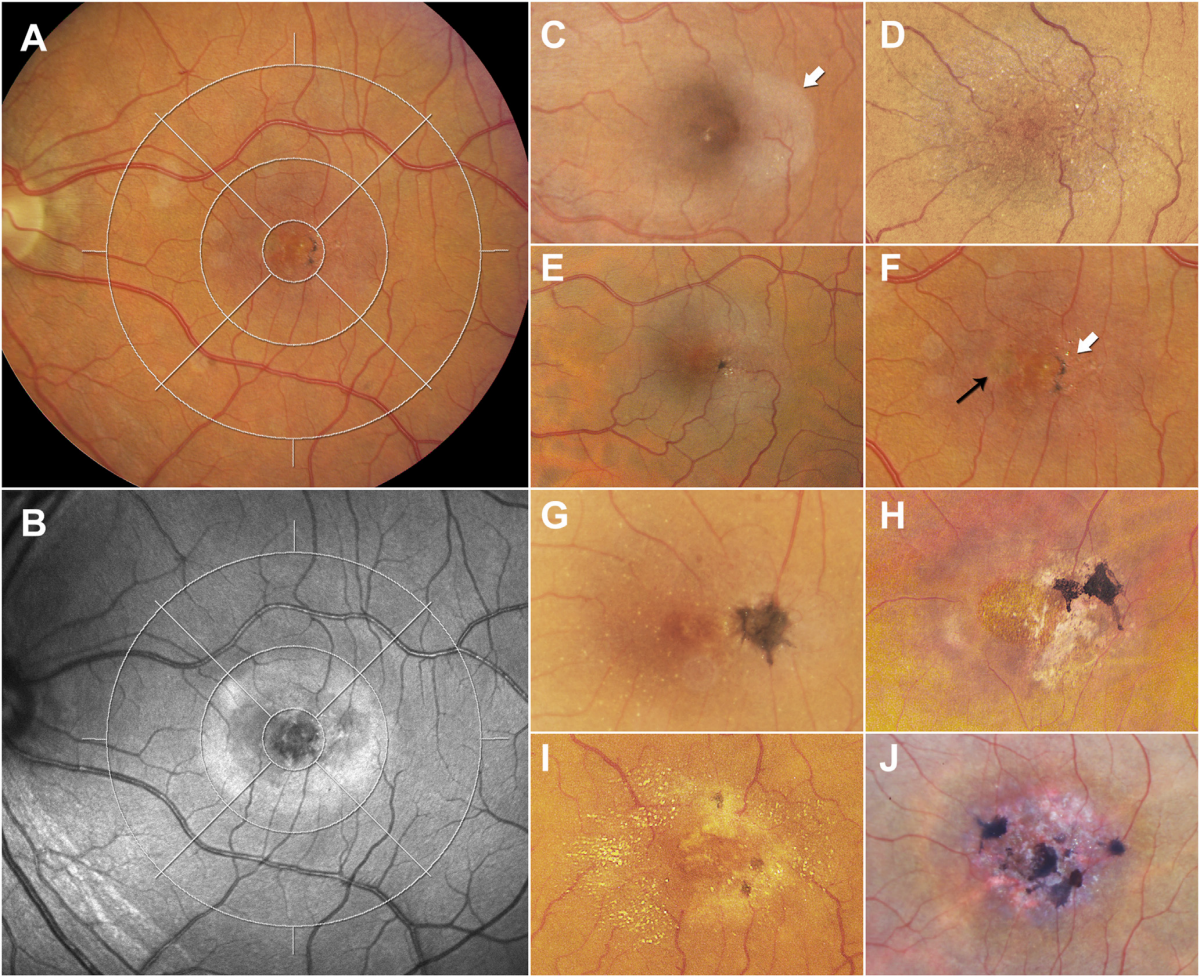
Color fundus (CF) image analysis (all left eyes), The presence and distribution of a loss of retinal transparency and of brown perivascular pigment were analyzed in CF images using an International Classification (IC) grid centered on the fovea. An IC grid consists of 3 concentric circles of 1000, 3000, and 6000 -μm diameter and 4 spokes dividing the outer circles into superior, nasal, inferior, and temporal subfields (**A** and **B**). A loss of retinal transparency (increased scatter) is also detectable in confocal scanning laser ophthalmoscopy images using short wavelength (blue) light (**B**). (**C**) demonstrates a loss of retinal transparency (white arrow) in the temporal inner subfield of the grid. (**D**) shows a loss of retinal transparency in all 4 quadrants of the middle ring of the IC grid, as well as superficial retinal crystals and microvascular anomalies in the temporal subfield. (**E**) shows a loss of retinal transparency, a few crystals and a small dark pigment associated with 2 converging venules on the boundary of the central and the temporal subfields. (**F**) demonstrates a bifocal pigmentation (white arrow) with an additional suspected pigmentation in the deeper layers of the retina on the nasal side of the foveal center (thin black arrow). (**G**) demonstrates a unifocal larger dense dark brown pigment plaque with tissue contraction demonstrated by straight radial and tortuous circumferential vessels. (**H–J**) demonstrate various stages of multifocal brown pigment that may also occur at the apparent foveal center.

Variables included in these analyses were both diagnostic of disease and may change over time, such as fluorescein leakage. In CF photographs, the presence and distribution of a loss of retinal transparency and perivascular pigmentary changes were assessed within the central 5 subfields of an International Classification grid^25^ centered on the fovea (Fig 1). Although loss of retinal transparency is better detected with the more recently available confocal BLR imaging (Fig 1A, B), for historical consistency, images of this modality were not included in the current grading.

**Figure 2:**
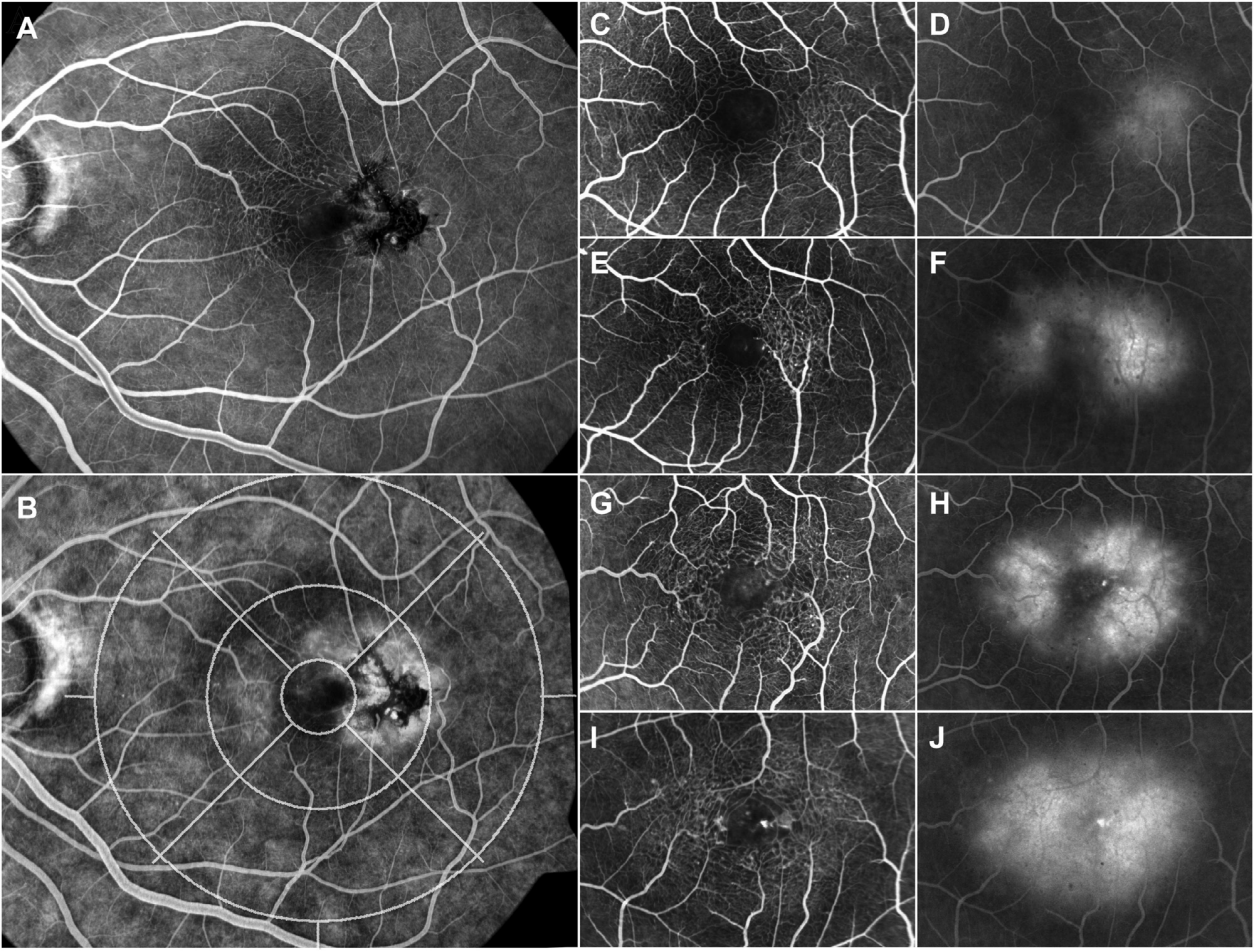
Analysis of fundus fluorescein angiographic (FFA) images (all left eyes). As in color fundus images, FFA images were analyzed using an International Classification (IC) grid. (**A**) shows an early phase of the angiogram (with only partial filling of the veins). At this phase small vessel anomalies are well visible. (**B**) shows a late phase angiogram, with the IC grid superimposed over the image. At this phase, the presence and distribution of leakage of the dye on the level of the deep capillary plexus and the retinal pigment epithelium are measured by grid subfield and by type of leakage (focal/diffuse/mixed). The type of leakage was determined based on whether the source of the leakage could be identified just after the transit of the dye through the capillaries. All cases presented in this figure represent focal leakage, although at the late phase all appear diffuse. Leakage may be present without clear visible vascular morphological anomalies, as in (**C, D**), nasal versus the temporal part of the perifovea. (**C, E, G, I**) demonstrate increasing involvement of the perifovea in early phase FFA images, whereas (**D, F, H, J**) show the same structures in late phase angiograms. In (**C**), slightly dilated capillaries are apparent on the temporal side of the fovea. In (**E**), dilated capillaries and dilated and blunted venules are apparent. In (**G**) dilated capillaries and a right-angle vein on the temporal side are evident. In (**I**) a full circle of involvement of dilated capillaries and corresponding late FFA leakage are demonstrated.

Fundus fluorescein angiographic images were analyzed by International Classification grid subfield for the presence and distribution of characteristic dilated, blunted and right-angle vessels (Fig 2), and for the distribution and type of (focal, diffuse, mixed) hyperfluorescence at the level of the deep capillary plexus or the retinal pigment epithelium.

**Figure 3:**
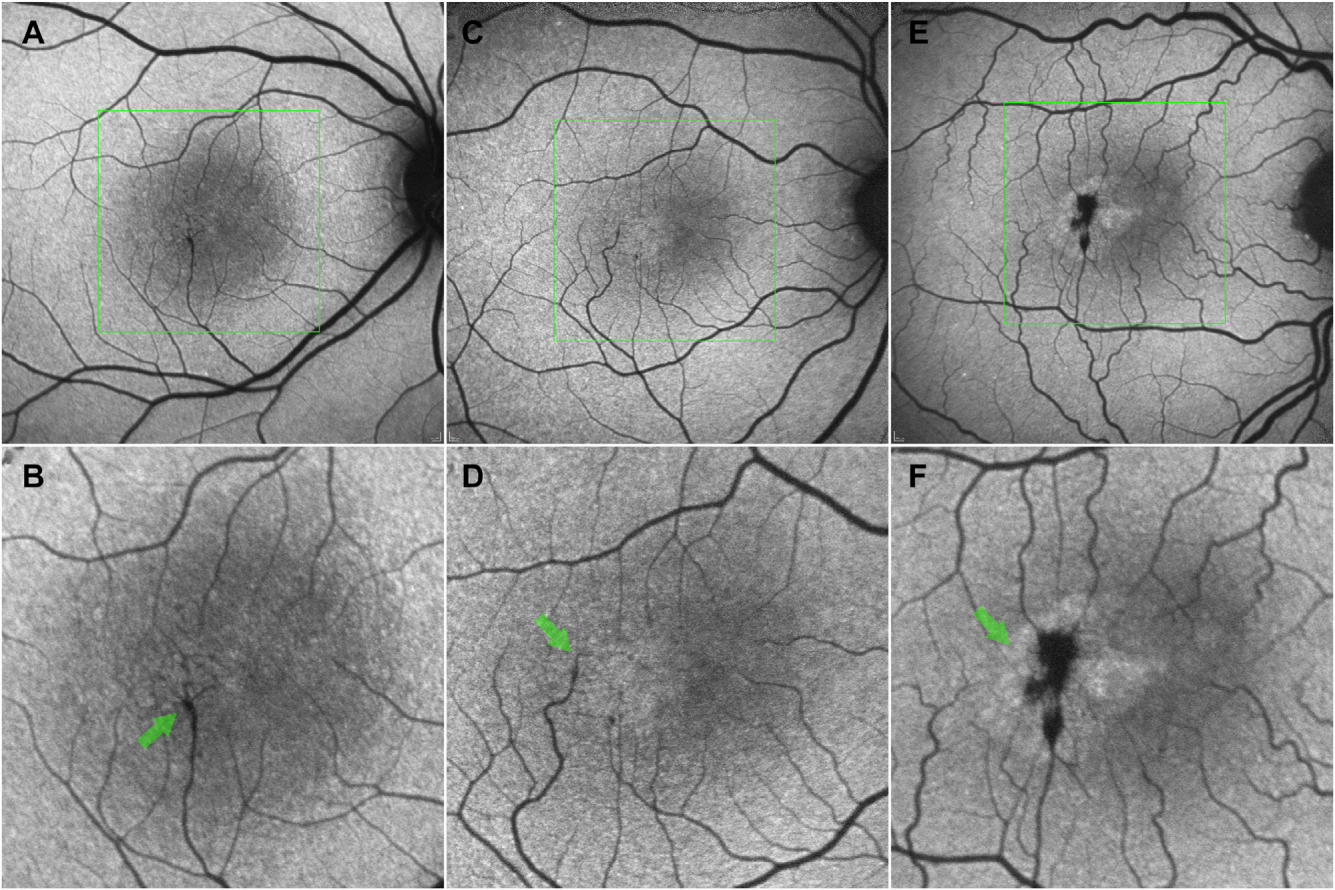
Fundus autofluorescence (FAF) image analysis (right eyes, top row standard FAF images, bottom row magnified central features for better visibility of detail), (**A**) and (**B**) show a loss of the normally present central luteal pigment peak, and a faintly increased autofluorescence (AF) temporal to the foveal center. The shadow gram of a blunted, right-angle vein is apparent temporal to the fovea (green arrow in 3B). In (**C**) and (**D**), a clear temporal wedge of increased AF (suggestive of loss of luteal pigment) is apparent with a fine right-angle vein (green arrow in [**D**]). In (**E**) and (**F**) the few vessels converge towards foci of decreased AF with straight end branches, against a background of increased AF temporal to the foveal center (green arrow in [**F**]), perivascular pigment plaque, and loss of luteal pigment.

The presence of direct and indirect signs of neovascularization (visible neovascularization lesion, hemorrhage, serous retinal detachment, or scar/fibrosis) was evaluated using both CF and fundus fluorescein angiographic images. Fundus autoflorescence images were also graded for the presence and location of vessel-adjacent focal hypo-autofluorescence (absent/present sign of perivascular pigment migration [Fig 3E, F], for the presence of blunted and right-angle vessels [Fig 3A–D], and for the presence of increased FAF signal in foveal area [a sign of luteal pigment loss/redistribution, Fig 3 A, B, E, F]).

**Figure 4:**
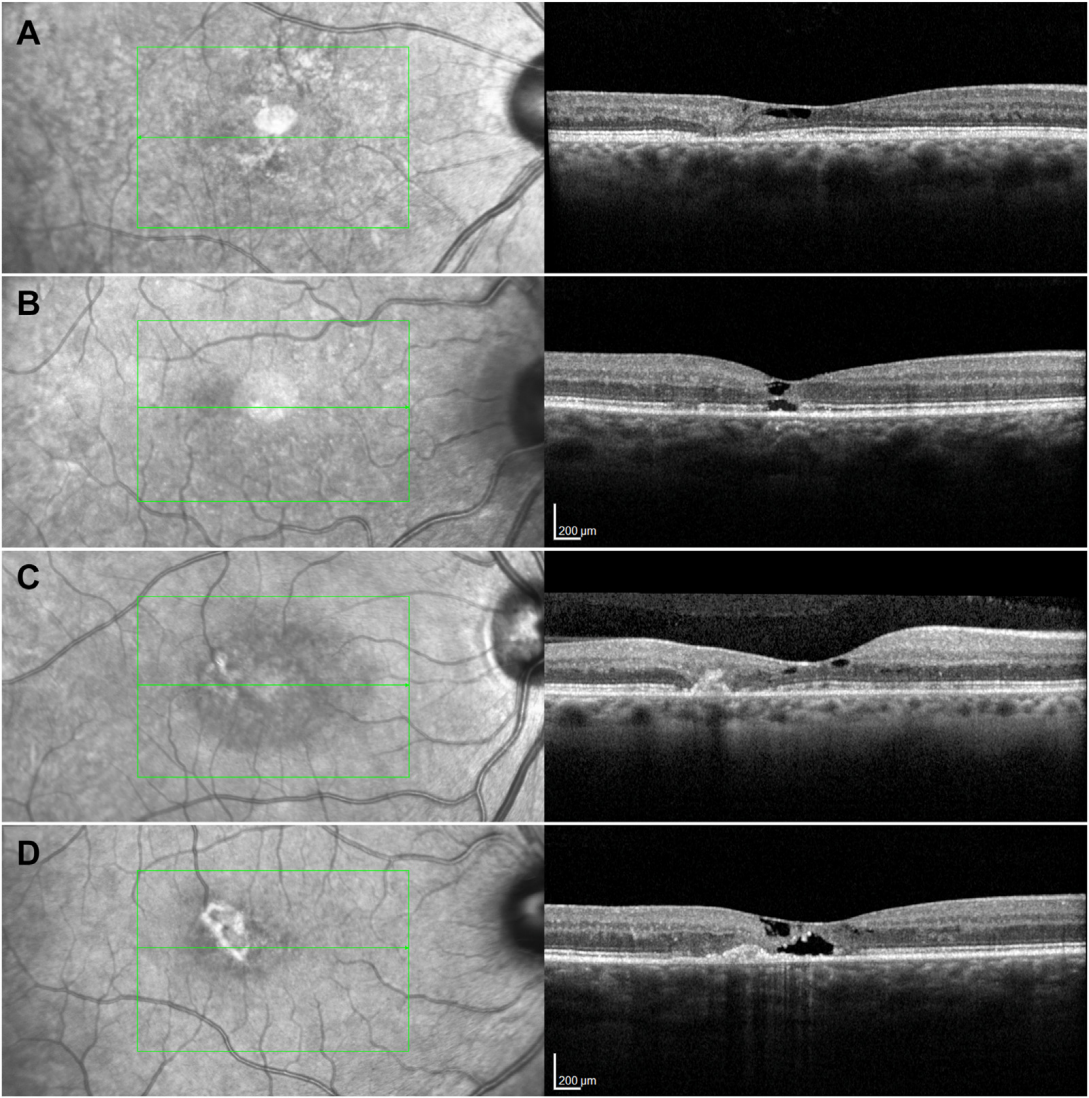
OCT features. (**A**) demonstrates a focal discontinuity in the ellipsoid zone (EZ) with a loss of the outer and a disorganization of the inner retinal layers, not involving the foveal center. A dark area just external to the retinal surface represents an inner retinal low-reflective space. (**B**) demonstrates a discontinuity of the EZ with smaller low reflective spaces in the outer and the inner retina and a slight hyper-reflectivity of the external limiting membrane. (**C**) shows discontinuity temporal to the fovea not involving the center of fovea, as well as a hyper-reflective lesion in the outer retina. (**D**) shows outer retinal hyper-reflectivity. OCT hyper-reflectivity is defined as mounds of hyper-reflective material extending internally from the retinal pigment epithelium. They are associated with linear vertical or oblique hyper-reflective streaks. They may or may not be associated with shadowing corresponding to the retinal pigment seen on color fundus or fundus autofluorescence images.

OCT images were evaluated for a discontinuity (break) in the EZ (absent, present non-central, and present central), a sign of photoreceptor degeneration, (Fig 4A–C) and for the presence of a hyper-reflectivity in the outer retina (which may be attributed to perivascular retinal pigment migration with or without neovascularization Fig 4D).

**Figure 5:**
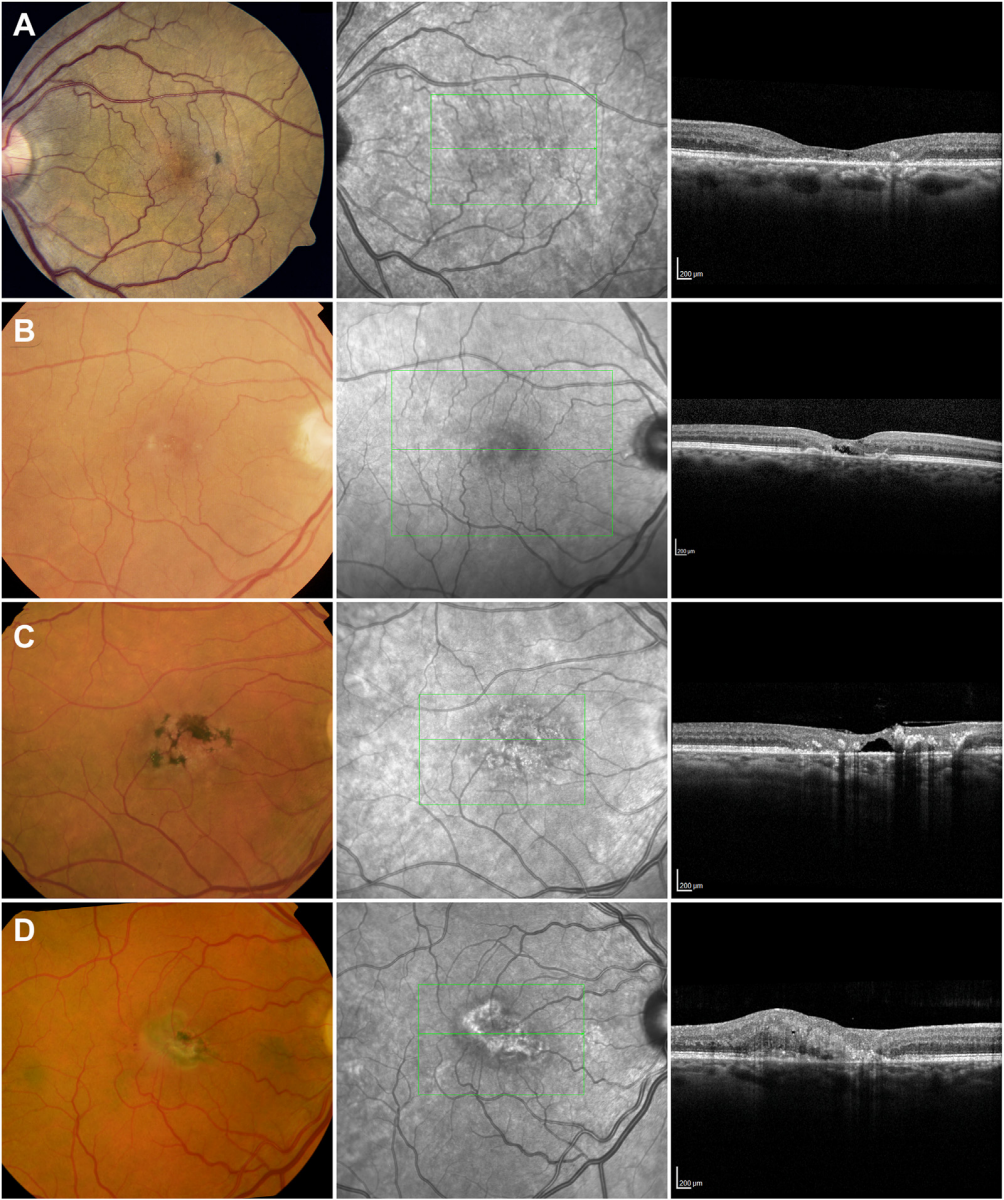
Representative images of grades 3 to 6 in color fundus (left column) and OCT B-scan images (right column). The middle column shows infrared images with green markers indicating the OCT grid placement and the position of the representative OCT B-scan within the scan volume. The top row (**A**) shows a small patch of noncentral pigment, a wide ellipsoid zone (EZ) break with a small island of photoreceptor at the center and no OCT hyper-reflectivity. The second row (**B**) demonstrates the presence of OCT hyper-reflectivity within an EZ break involving the foveal center, but no pigment. The third row (**C**) shows brown pigment also present in the central subfield of the International Classification grid, an extensive EZ break, with no exudative neovascularization. The bottom row (**D**) shows an active neovascular lesion with small patches of hemorrhage in the color image and retinal thickening, indistinct lesion boundaries, and intraretinal fluid in the OCT B-scan.

## Results

A total of 1733 participants from the NHOS and NHOR had images available for the classification development. A total of 1292 right eye images were used for the test dataset and 1302 left eye images were used for the validation data set. Additional validation was conducted on the last available image for 1733 right and left eyes, respectively, of natural history observation and NHOR participants. For the 1733 participants (687 males and 1046 females; mean age 60.8 years ± 9.7 years), a total of 755 participants (290 males and 465 females, age 60,7 ± 9.2 years) had ≥ 1 year of follow-up for the additional progression validation analyses.

The images were graded for the varying severity grade using the Gass-Blodi classification system (Table S2). Nearly 50% of the severity of eyes in the natural history study were in the grade 3 Gass classification (presence of right-angle veins) and 20% were in grade 4 (pigment present). The correlation of the 2 classification systems was also assessed in both the right eyes (Table S3) and the left eyes (Table S4).

After the completion of the initial classification development using CART (Fig S1), the following variables were found to be significant for progression to VA loss: EZ loss, pigmentary changes, and OCT hyper-reflectivity (Figure 5). These factors that had an impact on VA decline were placed into order with other factors, based upon the progression from good VA to poor VA at initial baseline for the right eye (Fig S1 and Table S1, Supplementary Materials on Results). As validation of this classification, the order of factors was then evaluated using the left eyes of the participants, again, ordering the factors based upon the progression from good VA to poor VA at baseline. Additional analyses were conducted using the same process of ordering factors according to diminishing VA for the last visit of the right eye and again for the left eye. All these results are demonstrated in the Table S1 in the Supplementary Materials on Results. All 4 analyses had placed these factors in the order that produced the final classification.

### The Classification of MacTel

Based on these analyses we propose a 7-grade classification of MacTel (Table 2). This classification is used to assess eyes that already have their diagnoses of MacTel confirmed by ocular imaging, particularly with OCT, FAF, BLR, and including fluorescein angiography and CF photographs. There are 7 grades with the initial grade (Grade 0) demonstrating only the key features that are diagnostic of MacTel and none of the 3 factors found to be significant for progression to VA loss (EZ loss, pigmentary changes, and OCT hyper-reflectivity). In the presence of a nonfoveolar EZ break (Grade 1), it is not surprising that the VA is unaffected; this begins in a noncentral location. However, when the EZ break affects the center of the fovea, the VA scores drop by almost 10 letters in grade 2 and 15 letters on average in Grade 3.^26^ The next big step for VA decline is heralded by the presence of the OCT hyper-reflectivity (Grade 4), followed by the presence of central pigmentary changes (Grade 5). The presence of pigment and exudative neovascularization result in the most severe grade of this classification (Grade 6).

**Table 2:** Classification of Macular Telangiectasia Type 2 (MacTel)

	Classification of Macular Telangiectasia Type 2 (MacTel Classification) (Following Diagnosis of MacTel Confirmed with OCT, Fluorescein Angiography, Fundus Autofluorescence, Blue-light Reflectance, or Color Fundus Photographs)
Grade	Description of Each Grade
0	No EZ Break/No Pigmentation/No OCT HR
1	Noncentral EZ Break/No pigment/No OCT HR
2	Central EZ break/No pigment/No OCT HR
3	Noncentral pigment/No, non-central, or central EZ/No OCT HR
4	OCT HR/EZ break (either central or noncentral)/No pigment
5	Central pigment/no exudative neovascularization/EZ present or not gradable
6	Neovascularization (exudative) ± central pigment

Noncentral is equivalent to having the central subfield of the International Classification grid be unaffected, free of lesion.

EZ = ellipsoid zone; HR = hyper-reflectivity.

### Follow-Up Analyses Using the Mac Tel Classification System

In the analyses of the 755 participants who had sufficient follow-up data, using the Generalized Estimating Equation regression models, we evaluated the annual relative risk of progression over a period of 5 years for the following outcomes: progression to ≥ 5 letter loss, ≥ 10 letter loss from baseline (Figs S2 and S3), 1 or 2 step progression (Figs S4 and S5), and progression to steps 4 and 5 (Figs S6 and S7). For example, an increasing relative risk of progression with time is expected. Most VA decline reached almost 15 letters when progression to level of 3 occurred; thus, the rate of progression to VA loss is much less in those in grade 4, as seen on Figure S3.

## Discussion

In this analysis we have used data from MacTel Study participants that incorporates state-of-the-art imaging modalities, including SD-OCT. The CART analyses demonstrated 3 important features key to the construction of this classification: OCT hyper-reflectivity, pigment, and EZ discontinuity (break). The primary target of interest for the algorithm development by CART was the decline in BCVA. This classification was built using the data from the right eye and targeting the loss of VA over time. The validation studies using the left eyes of the NHOS and both eyes from the NHOS last study visit provided further evidence of support for this classification.

It is not surprising that this classification shares features with the Gass classification (Table 1), including features of the most severe end of the scale, the presence of pigmentation and exudative neovascularization, as these events caused the most severe loss of vision. The direct comparison of the 2 systems (Tables S3 and S4) also demonstrated the exact agreement between those with pigmentation and exudative neovascularization. In addition, the first grade of the current classification does not include any OCT abnormalities in the EZ layer. These early grade patients only manifest changes typical for a diagnosis of MacTel (fluorescein angiographic leakage, the presence of retinal opacification, blunted or right-angle veins, and typical BLR and FAF imaging). The correlation between these earlier grades of the MacTel Classification with those of the Gass-Blodi classification was poor (Tables S3 and S4) as the 2 systems used very different variables. The variables in the Gass-Blodi system are descriptive factors that deal mostly with the vascular component of the disease while those in the MacTel Classification used structural OCT changes that translated to VA loss (Table S1).

The subsequent grades of this classification demonstrate structural changes that result in VA loss—not surprising, as we found correlations of the structural changes with VA changes.^26^ The first is the EZ discontinuity (break) which has little impact on VA when present in the noncentral area. However, once the EZ break involves the center of the fovea, VA declines. In the past, we reported that a deep learning algorithm can be used to measure the volumes of the cavitations,^27^ hyporeflective space that does not correlate with fluorescein leakage. These cavitation volumes are associated with VA loss, and it appears that these cavitations precede the EZ loss, especially as it encroaches on the foveal center.^28^

The next decrease in VA occurs with the presence of noncentral pigment regardless of the EZ loss status. This signifies a progression with worsening of VA. Further VA loss is evident with the presence of OCT hyper-reflectivity. It should be noted that both grades 3 and 4 are closely related, as often the OCT hyper-reflectivity may occur prior to the formation of pigment. This classification is built based upon VA decrease, thus the eye is not expected to go through each of the severity grades. The authors have also examined patients who progressed directly from grade 1 or 2 to exudative neovascularization, a severe end of the classification. Again, this classification is built upon the decrease in VA.

The OCT hyper-reflectivity is the next grade of severity accompanied with VA loss; this was evaluated by the reading center early in the history of the NHOR study when the composition of these lesions was not well understood. Fluorescein angiography of such eyes typically demonstrated the usual perifoveal leakage from the dilated telangiectatic vessels. With the more recent use of OCT angiography (OCTA), these hyper-reflective lesions seen on OCT are now considered to be intraretinal, nonexudative neovascularization. A longitudinal series of 12 patients with OCTA showed these represented retinal-choroidal anastomosis following the subsidence of the retina, which is defined as the loss of the outer nuclear layer with the retina sinking towards the retinal pigment epithelium.^29^ The authors considered these lesions to be retinal-choroidal anastomosis (known as type 3 macular neovascularization) colocalized to the area of the OCT hyper-reflectivity. However, other investigators have considered these images only as outer intraretinal neovascularization and not retinal-choroidal anastomoses.^13^ These lesions were not associated with retinal thickening and fluorescein angiography leakage (other than the typical leakage found with the perifoveal telangiectasis), lipid, hemorrhage, or any other fluid exudation along with no evidence of subretinal/subretinal pigment epithelial neovascularization. The OCT hyper-reflectivity has also been well described recently by other MacTel Project investigators.^13^^,^^15^ It is reassuring that our findings agree with other groups, reinforcing the relevance of these imaging modalities to disease grade and risk of progression to vision loss.^30^

In order to provide a “simple scale” for the clinician for practical use in the clinic while examining patients, this classification can be reduced to the main features for the following grades: 0 to 2 means no EZ loss, noncentral EZ loss, and central EZ loss, respectively; grade 3 heralds the presence of **noncentral pigment,** grade 4 is the presence of **OCT hyper-reflectivity**, grade 5 is presence of **central pigment**, and finally grade 6 is the most severe end with the development of **exudative neovascularization** (Table 3). Each of these grades was represented by key lesions with the corresponding decline in the VA to the most severe grade of this simple MacTel classification.

**Table 3:** Simple Classification of Macular Telangiectasia Type 2. (Simple MacTel Classification)

	Simple MacTel Classification
Grade	Essential factor of Each Grade
0	No lesions
1	Noncentral ellipsoid zone (EZ) Break
2	Central EZ break
3	Noncentral pigment
4	OCT hyper-reflectivity
5	Central pigment
6	Neovascularization (exudative)

EZ = Ellipsoid zone; MacTel = Macular Telangiectasia Type 2.

There are limitations to this study. An important limitation is the use of the left eye to validate an algorithm which was developed using the right eye of the same participants. This may be a disadvantage because eyes are highly correlated. Ideally, a training set, a validation set, and a test set would be conducted using both eyes of this dataset and divided by participants. It is also important to be able to validate this algorithm on an entirely separate dataset involving different participants. Such an external validation data set of a longitudinal assessment of cohort of participants with the diagnosis of this somewhat rare disease, MacTel, is not available. However, a limited dataset for the future may be the sham-treated participants who are currently enrolled in our Phase II^31^ and III clinical trials of ciliary neurotrophic factor studies (ClinicalTrials.gov identifier (NCT number: NCT03319849 and NCT03316300), which we expect to complete follow-up in the last quarter of 2022. These study participants, however, all have EZ loss to be eligible for the clinical trial and would only validate the more severe end of the classification with relatively short follow-up in ≥ 200 participants.

Another limitation includes the potential for selection bias for enrollment at early stages of the disease. However, with NHOR, the spread of disease included fairly equal proportions with early to later stages of the disease. In short of doing a population-based study, which is not feasible in this case, there may be concerns of selection bias, but this may be low in this instance.

A final limitation of the study is that an important technology, OCTA, was not included in the natural history study of this condition because it was not yet available at the beginning of the study. For this neurovascular-glial condition, the use of OCTA, as noted previously, has proven to be essential to diagnose both the intraretinal neovascularization (type 3 macular neovascularization) and typical macular neovascularization (type 2). The technology is relatively new, and the data are accumulating for MacTel and the development of neovascularization.

This classification can indeed be influenced again by future imaging that has not yet developed or is not yet commonly used. Experimentally, the fluorescence lifetime imaging ophthalmoscopy (FLIO) has been used to detect early grades of MacTel.^18^ The FLIO is a noninvasive method of imaging modality that provides additional information regarding the autofluorescence of the retina. Detecting FAF lifetimes allows the detection of subtle changes that may be found, especially in the early grades of the disease. In fact, children who are considered unaffected may be detected prior to the classic onset of the disease with the use of FLIO.^32^ Other researchers demonstrated that the FLIO also provides information regarding the macular pigment and possibly photoreceptor loss.^33^ They also concluded that FLIO correlates with disease severity. Similarly, Sauer et al. conducted a longitudinal study of FLIO in persons with MacTel and found that autofluorescence lifetimes slowly prolonged over time with very specific patterns.^34^ Although such data on this condition may be informative in future developments of the MacTel classification, it will depend on how FLIO will be incorporated into clinical care. Currently, it is highly experimental and is limited by the small number of instruments available for testing. This may or may not change in the future.

This current classification is considered a “living document” in that newer technology and additional data may provide further findings for yet another revision of the MacTel Classification. We anticipate that with the development of greater resolution of the ocular imaging, potential contributions of the FLIO images and other imaging modalities such as OCTA and adaptive optics, we will gain further insight into MacTel and changes in this classification are inevitable. This newly developed classification may provide a framework to communicate effectively to other researchers, physicians taking care of the patients, and to the patients directly.
