# Supplementary material for: Macular Telangiectasia Type 2: A Classification System Using MultiModal Imaging MacTel Project Report Number 10
Source: Ophthalmol Sci. 2022 Dec 8;3(2):100261. doi: 10.1016/j.xops.2022.100261 (PMC9944556; doi:10.1016/j.xops.2022.100261)
Supplement: Figs S1–S7 [file mmc1.pdf]

Supplementary Materials (Figures)

e-Figure 1: Tree Structure Analyses of Progression of Visual Acuity Decrease in Macular Telangiectasia Type 2 (MacTel)

e-Figure 2 to 7: Visual Acuity loss across the scale and the Relative risk of progression along the scale over 5 years of follow-up:

eFigure 2: 5 or more letter loss across all stages of the Mac Tel Classification over a period of 5 years.

eFigure 3: 10 or more letter loss across all stages of the Mac Tel Classification over a period of 5 years.

eFigure 4: 1-step progression along all stages of the Mac Tel Classification over a period of 5 years.

eFigure 5: 2-step progression along all stages of the Mac Tel Classification over a period of 5 years.

eFigure 6: Progression to step 4 or greater along all stages of the Mac Tel Classification over a period of 5 years.

eFigure 7: Progression to step 5 or greater along all stages of the Mac Tel Classification over a period of 5 years.

e-Figure 1:

Tree Structure Analyses of Progression of Visual Acuity Decrease in Macular Telangiectasia Type 2 (MacTel)

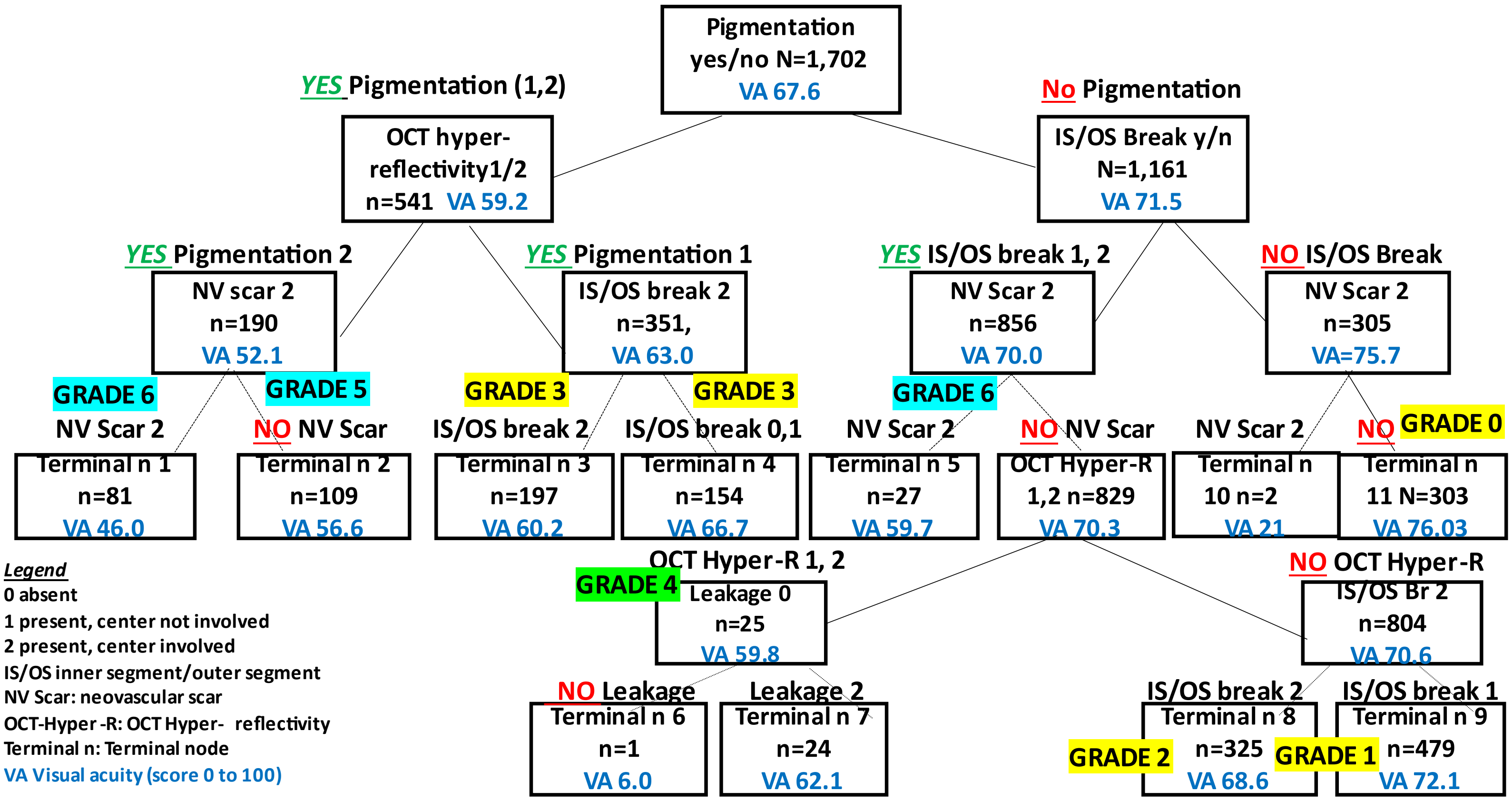

e-Figure 2 to 7: Visual Acuity loss across the scale and the Relative risk of progression along the scale over 5 years of follow-up

Figure e2: 5 or more letter loss across all stages of the Mac Tel Classification over a period of 5 years.

Figure e3: 10 or more letter loss across all stages of the Mac Tel Classification over a period of 5 years.

Figure e4: 1-step progression along all stages of the Mac Tel Classification over a period of 5 years.

Figure e5: 2-step progression along all stages of the Mac Tel Classification over a period of 5 years.

Figure e6: Progression to step 4 or greater along all stages of the Mac Tel Classification over a period of 5 years.

Figure e7: Progression to step 5 or greater along all stages of the Mac Tel Classification over a period of 5 years.

e-Figure 2

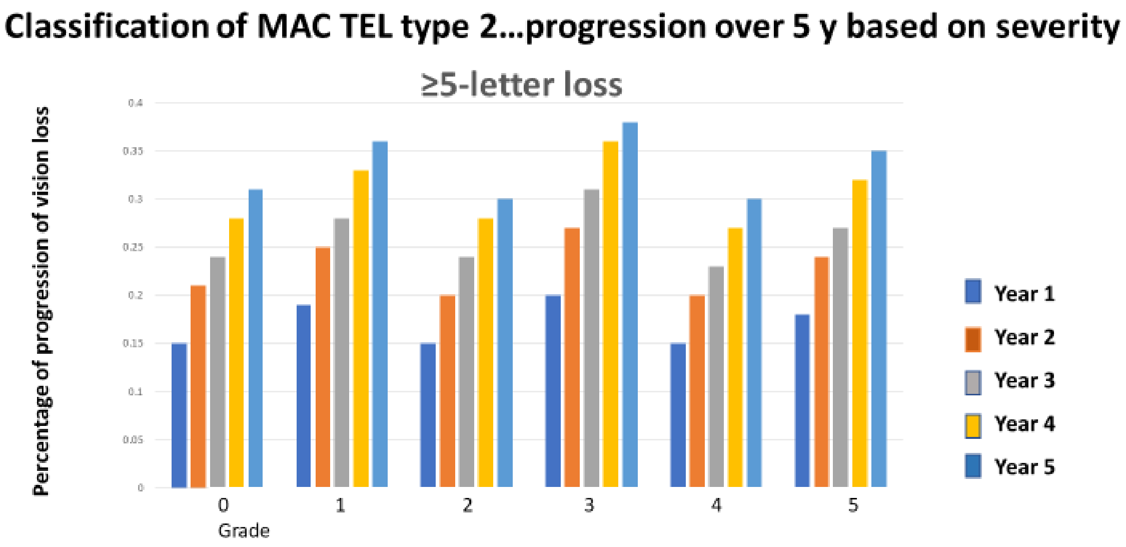

Progression to loss of 5 or more letters for each year of follow-up, based upon the baseline severity of Mac Tel (grade)

e-Figure 3

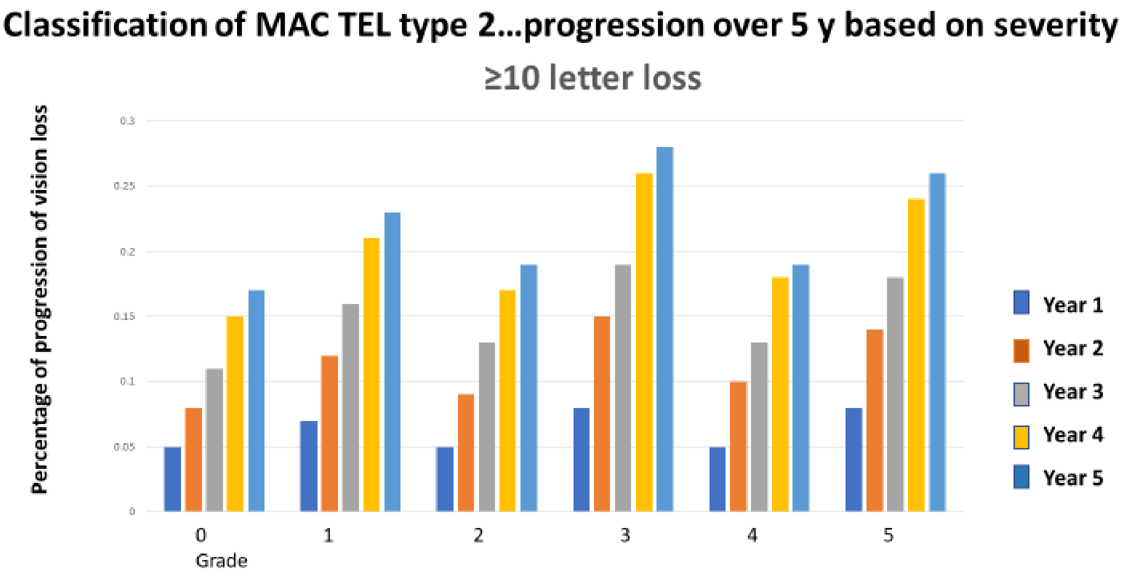

Progression to loss of 10 or more letters for each year of follow-up, based upon the baseline severity of Mac Tel (grade)

e-Figure 4

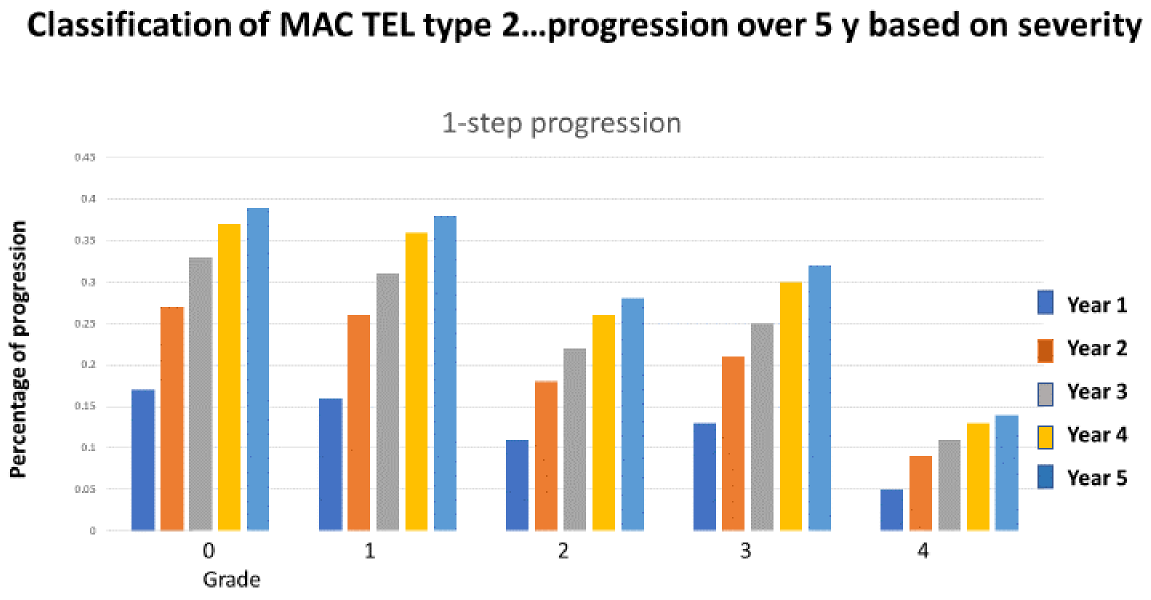

Progression of 1 step along the severity scale for each year of follow-up, based upon the baseline severity of Mac Tel (grade)

e-Figure 5

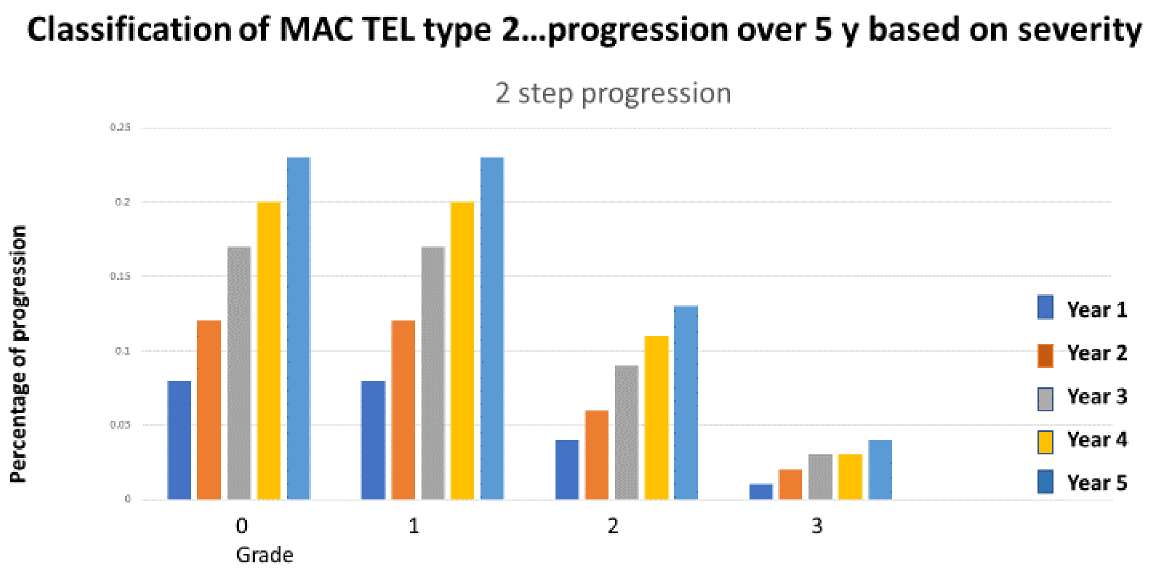

Progression of 2 steps along the severity scale for each year of follow-up, based upon the baseline severity of Mac Tel (grade)

e-Figure 6

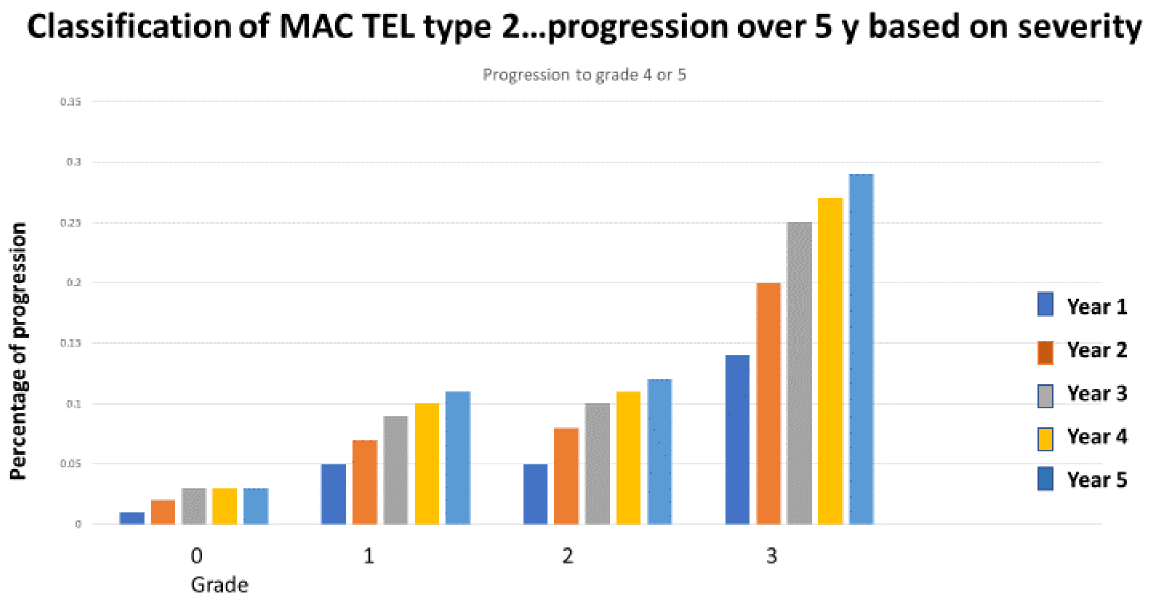

Progression to severity level of 4 or 5 along the Mac Tel severity scale for each year of follow-up, based upon the baseline severity of Mac Tel (grade)

e-Figure 7

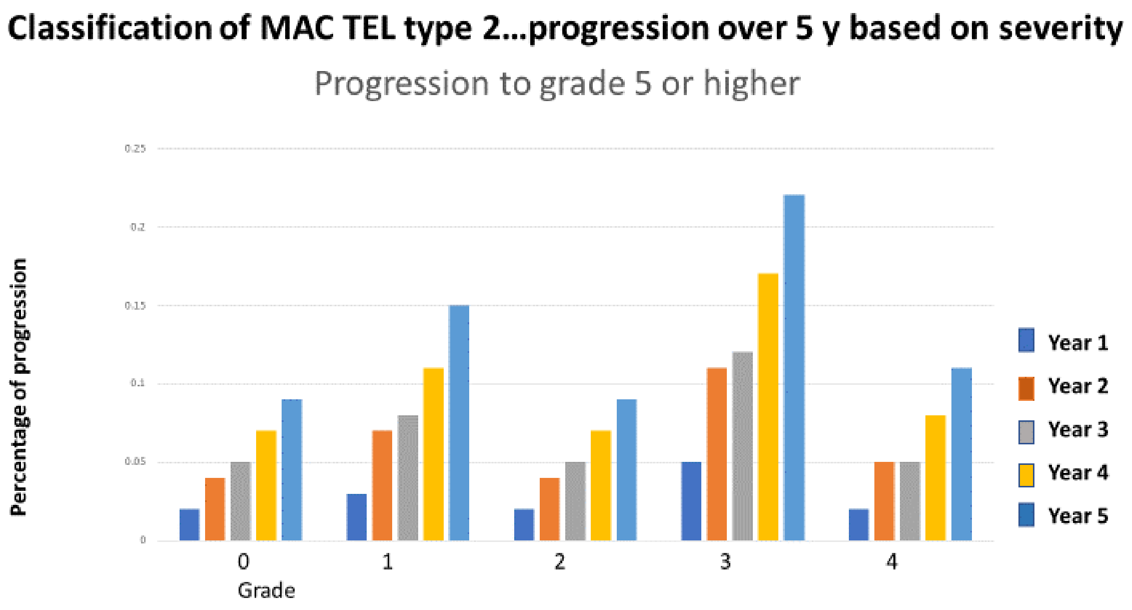

Progression to severity level of 4 or 5 along the Mac Tel severity scale for each year of follow-up, based upon the baseline severity of Mac Tel (grade)
