# Supplementary material for: Macular Telangiectasia Type 2: A Classification System Using MultiModal Imaging MacTel Project Report Number 10
Source: Ophthalmol Sci. 2022 Dec 8;3(2):100261. doi: 10.1016/j.xops.2022.100261 (PMC9944556; doi:10.1016/j.xops.2022.100261)
Supplement: Supplementary Materials [file mmc2.pdf]

Supplementary Materials (Methods)

Decision Tree Methodology

Decision Tree Methodology

The Decision Tree method is a data mining method for establishing classification systems based on multiple covariates. Can also be used for developing prediction algorithms for a target variable of interest. The methodology classifies a population into branch-like segments that construct an inverted tree with a root node, internal nodes, and leaf nodes. The algorithm is non-parametric and thus can be used on any type of data as it does not impose a complicated parametric structure. It can also deal with large, complicated data sets. When using this methodology best to have 2 datasets: 1) training dataset and 2) validation dataset. For MacTel can use for example left eyes as the training dataset and right eyes as the validation dataset. The training dataset will be used to build the decision tree model and the validation data set can be used to decide on the appropriate tree size needed to achieve the optimal final model.

Concepts:

Nodes - There are three types of nodes:

- Root node: also called a decision node, represents a choice that will result in the subdivision of all records into two or more mutually exclusive subsets
- Internal nodes: also called chance nodes, represent one of the possible choices available at that point in the tree structure; the top edge of the node is connected to its parent node and the bottom edge is connected to its child nodes or leaf nodes
- Leaf nodes: also called end notes, represent the final result of a combination of decisions or events.

Branches – represent chance outcomes or occurrences that emanate from root nodes and internal nodes. A decision tree model is formed using a hierarchy of branches. Each path from the root node through internal nodes to a leaf node represents a classification decision rule. These decision tree pathways can also be represented as “if-then” rules. Example – “if condition 1 and condition 2 and condition XXX occur, then outcome Y occurs.”

Splitting - Only input variables related to the target variable are used to split parent nodes into purer child nodes of the target variable. Both discrete input variables and continuous input variables (which are collapsed into two or more categories) can be used. When building the model one must first identify the most important input variables, and then split records at the root node and at subsequent internal nodes into two or more categories or ‘bins’ based on the status of these variables. Characteristics that are related to the degree of ‘purity’ of the resultant child nodes (i. e. the proportion with the target condition) are used to choose between different potential input variables. This splitting procedure continues until pre-determined homogeneity or stopping criteria are met. In most cases, not all potential input variables will be used to build the decision tree model and, in some cases, a specific input variable may be used multiple times at different levels of the decision tree.
