# Supplementary material for: Macular Telangiectasia Type 2: A Classification System Using MultiModal Imaging MacTel Project Report Number 10
Source: Ophthalmol Sci. 2022 Dec 8;3(2):100261. doi: 10.1016/j.xops.2022.100261 (PMC9944556; doi:10.1016/j.xops.2022.100261)
Supplement: Tables S1–S4 [file mmc3.pdf]

Supplementary Materials (Tables)

eTable 1: Classification of Macular Telangiectasia type 2 by Visual Acuity Score (0 to 100) at baseline and for the last study visit for right, left eyes respectively

eTable 2: The proportion of eyes of NHOR participants stratified by the levels of severity by the Gass/Blodi Classification

eTable 3: Correlation of Gass-Blodi classification with Mac Tel Classification in the right eyes of NHOR participants

eTable 4: Correlation of Gass-Blodi classification with Mac Tel Classification in the left eyes of NHOR participants

eTable 1

Classification of Macular Telangiectasia type 2 by Visual Acuity Score (0 to 100) at baseline and for the last study visit for right, left eyes respectively.

	**Classification of Macular Telangiectasia Type 2 (MacTel)**	**Visual Acuity Scores at baseline (BL) and at last study visits:** OD right, OS left			
Stage	**Description of Each Grade** (all eyes have been confirmed to have MacTel with OCT, FA, FAF, blue light reflectance or color fundus photos)	BL OD	BL OS	Last OD	Last OS
0	No Ellipsoid zone (EZ) Break/No Pigmentation/No OCT Hyper-reflectivity (HR)	75.7	78.3	76.1	78.6
1	Noncentral EZ Break/No pigment/No Oct HR	72.1	73.0	71.6	72.7
2	Central EZ break/No pigment/No OCT HR	68.6	70.3	67.4	69.5
3	Noncentral pigment/No, noncentral or central EZ loss/No OCT HR	60.2	64.5	63.9	67.7
4	OCT HR/EZ break (either central or noncentral)/no pigment	59.8	62.9	61.6	63.2
5	Central pigment/no exudative neovascularization/EZ present or not gradable	56.6	57.9	57.1	57.3
6	Neovascularization (exudative) ± central pigment	45.9	53.2	44.9	51.6

eTable 2 The Proportion of Eyes of NHOR participants Stratified by the Levels of Severity of the Gass/Blodi Classification

Right eyes				Left eyes		
Stage of Gass/Blodi Classification	N	%		Stage of Gass/Blodi Classification	N	%
1	138	8.2		1	160	9.5
2	184	10.9		2	211	12.5
3	795	47.1		3	808	47.9
4	399	23.7		4	372	22.1
5	171	10.1		5	135	8.0

e-Table 3:
Correlation of Gass-Blodi Classification with Mac Tel Classification in the Right Eyes of **NHOR** Participants

Revised Simple MacTel Classification

0. No abnormalities
1. Non-central EZ loss
2. Central EZ loss
3. Non central pigment
4. OCT Hyperreflectivity
5. Central pigment
6. Neovascularization

(red circle) Exact agreement between the two classificaitons

Revised MacTel Classification	Gass-Blodi Classification				
	1	2	3	4	5
0	35	68	195	0	0
1	10	54	296	38	0
2	10	51	262	0	0
3	0	0	0	(58)	0
4	10	2	22	0	0
5	0	0	0	(108)	0
6	0	0	0	0	(81)

Gass Blodi Classification

1. No abnormality
2. Retinal Opacities
3, Right angle vessels
4. Pigment
5. Neovascularization

e-Table 4:
Correlation of Gass-Blodi Classification with Mac Tel Classification in the Left Eyes of **NHOR** Participants

Revised Simple MacTel Classification

0. No abnormalities
1. Non-central EZ loss
2. Central EZ loss
3. Non central pigment
4. OCT Hyperreflectivity
5. Central pigment
6. Neovascularization

(red circle) Exact agreement between the two classificaitons

Revised MacTel Classification	Gass-Blodi Classification				
	1	2	3	4	5
0	50	94	234	0	0
1	12	70	278	24	0
2	13	41	234	0	0
3	0	0	0	(41)	0
4	0	3	33	0	0
5	0	0	0	(112)	0
6	0	0	0	0	(63)

Gass Blodi Classification

1. No abnormality
2. Retinal Opacities
3, Right angle vessels
4. Pigment
5. Neovascularization
